# Reproductive effects of subchronic exposure to acetamiprid in male rats

**DOI:** 10.1038/s41598-020-65887-0

**Published:** 2020-06-02

**Authors:** Emre Yağmur Arıcan, Damla Gökçeoğlu Kayalı, Bahar Ulus Karaca, Tuğçe Boran, Narin Öztürk, Alper Okyar, Feriha Ercan, Gül Özhan

**Affiliations:** 1 Istanbul University, Faculty of Pharmacy, Department of Pharmaceutical Toxicology, 34116 Istanbul, Turkey; 2 Marmara University, Faculty of Medicine, Department of Histology and Embryology, 34854 Istanbul, Turkey; 3 Istanbul University, Faculty of Pharmacy, Department of Pharmacology, Istanbul, 34116 Turkey

**Keywords:** Environmental sciences, Risk factors

## Abstract

Acetamiprid, a selective agonist of nicotinic acetylcholine recetors, is one of the most widely used neonicotinoids. There is limited data about toxicity of acetamiprid on male reproductive system. Therefore, the study aimed to investigate the reproductive toxic potential of acetamiprid in male rats orally treated with acetamiprid with low (12.5 mg/kg) medium (25 mg/kg) or high dose (35 mg/kg) for 90 days. According to our results, sperm concentration and plasma testosterone levels decreased in dose dependent manner. Gonadotropin-releasing hormone (GnRH), follicle-stimulating hormeone (FSH), luteinizing hormone (LH) levels increased at low and medium dose groups and acetamiprid caused lipid peroxidation and glutathione (GSH) depletion in the testes. Histologic examinations revealed that acetamiprid induced apoptosis in medium and high dose groups and proliferation index dramatically decreased in high dose group. In conclusion, acetamiprid caused toxicity on male reproductive system in the high dose. The mechanism of the toxic effect may be associated with oxidative stress, hormonal disruptions and apoptosis.

## Introduction

Neonicotinoids are new class of insecticides that act as selective nicotinic acetylcholine receptor (nAChR) agonist selectively in central nervous system of insects^1^. Acetamiprid, one of the neonicotinoid insecticides, is commonly used for agricultural and domestic purposes against a large variety of insects^2,3^. Acetamiprid has been reported to accumulate in plants and contaminate water and this can pose a potential risk for human health^4,5^^.^

Acetamiprid is absorbed easily after oral administration, and it is determined at the highest concentration in the liver, kidney, adrenal and thyroid glands^6^. Some researchers showed that acetamiprid caused toxic effects on several organ systems, including the nervous, respiratory, and immune systems in the experimental models^7–9^. Furthermore, it has been reported acute poisoning cases after ingestion of acetamiprid in humans^10,11^. Acetamiprid has also been reported to induce reproductive toxicity in different species^12,13^. The cross-sectional epidemiological study which was conducted in Kavar, (Iran) showed acetamiprid reduced the number of sperm in farmers who exposed to acetamiprid^14^.

As the use of acetamiprid is increasing, it is very important to identify the toxicity of acetamiprid. Additionally, acetamiprid can be used in combinations with other insecticide because of that, toxic effects and doses of acetamiprid are needed to elucidate well by chronic and subchronic toxicity studies. Acetamiprid has been shown toxic effects on many organs and systems. However, there is no satisfied information on the toxicity potential of acetamiprid on male reproductive system. In this study, it was aimed to examine the effects of acetamiprid on reproductive function of male rats in terms of oxidative stress, apoptosis, hormonal disruptions and histopathological changes.

## Results

### Effect of acetamiprid on body and testicular weights

Liver steatosis and slowness of the movements were observed at the high dose group. However, it was not found any differences in the food consumption of the groups. The body weights of control group were increased 2.14% comparing with first day of the study (*p* > 0.05). At the end of the 90^th^ day, the body weights were decreased nonsignificantly 1.98, 1.60 and 3.30% for low-, medium- and high-dose groups, respectively, compared to the beginning of the experiment. Similarly, there was no significant change in the testicular weights (*p* > 0.05) as compared to the control group (Table 1).

**Table 1:** Effect of oral acetamiprid on body weights and testicular weights of Sprague Dawley rats.

	Control group (n:10)	Low dose group (n:11)	Medium dose group (n:12)	High dose group (n:12)
Body weights (g)				
Day 1	307.60 ± 1.46	329.43 ± 2.65	341.93 ± 3.43	326.60 ± 2.34
Day 28	310.25 ± 1.65	326.45 ± 2.98	332.74 ± 2.76	322.15 ± 1.89
Day 90	314.18 ± 1.60	322.89 ± 3.55	336.46 ± 3.89	315.82 ± 2.59
Testicular weights (g) Day 90	1.50 ± 0.05	1.60 ± 0.03	1.55 ± 0.02	1.56 ± 0.05

Data were shown as mean ± standard error of the means (SEM). *p < 0.05, **p < 0.005, ***p < 0.0005.

### Effect of acetamiprid on sperm count and morphology

Sperm count and morphology evaluations were performed according to the World Health Organization (WHO) guideline^15^. The number of sperm was significantly decreased in the medium- and high-dose groups (*p* ≤ 0.004) as compared to the control group (Table 2). However normal sperm count and abnormal sperm count did not show any significant difference among the treatment groups. In the abnormal sperm morphology evaluations, significantly increase in the flattened headed sperm was observed in the high dose treatment group (*p* = 0.046).

**Table 2:** Evaluations of sperm morphology and counts in the Sprague Dawley rats treated orally with acetamiprid. Data were shown as mean ±  standard error of the means (SEM).

Sperm count (x10^6^/mL)				
	Control group (n:10)	Low dose group (n:11)	Medium dose group (n:12)	High dose group (n:12)
Sperm count	12.1 ± 0.4	11.4 ± 0.7	9.5 ± 0.5**	8.6 ± 0.4***
Sperm morphology (in 200 sperm)				
	**Control group (n:10)**	**Low dose group (n:11)**	**Medium dose group (n:12)**	**High dose group (n:12)**
	**Normal sperm count (mean ± SEM)**			
Normal	173.2 ± 3.8	174.0 ± 7.5	178.0 ± 3.5	163.6 ± 10.0
	**Sperm count with abnormalities (mean ± SEM)**			
Headless sperm	7.3 ± 0.7	5.3 ± 1.7	5.0 ± 1.3	10.6 ± 2.7
Detached head	13.1 ± 0.7	13.6 ± 1.7	10.3 ± 1.3	15.8 ± 2.7
Flattened head	1.5 ± 0.3	1.5 ± 0.3	1.2 ± 0.2	3.6 ± 1.2*
Pinhead	0.1 ± 0.1	0.3 ± 0.2	0.3 ± 0.1	0.3 ± 0.2
Bent neck	2.6 ± 0.3	2.7 ± 0.8	2.7 ± 0.6	3.7 ± 0.7
Bent tail	1.1 ± 0.2	1.2 ± 0.6	1.4 ± 0.5	1.1 ± 0.3
Coiled tail	0.7 ± 0.2	1.2 ± 0.4	0.9 ± 0.6	0.9 ± 0.3
Multiple abnomalities	0.3 ± 0.1	0.3 ± 0.1	0.3 ± 0.1	0.4 ± 0.2
Total sperm count with abnormalities	26.8 ± 3.9	26.0 ± 7.5	22.0 ± 3.5	36.4 ± 10.0
Percentage of the sperm with abnormalities (%)	13.4 ± 2.0	13.0 ± 3.8	11.0 ± 1.7	18.2 ± 5.0
Percentage of the normal sperm (%)	86.6 ± 2.0	87.0 ± 3.8	89.0 ± 1.7	81.8 ± 5.0

*p < 0.05, **p < 0.005, ***p < 0.0005.

### Effect of acetamiprid on hormone levels and oxidative stress parameters

The plasma testosterone (≥ 23%) and cholesterol (≥ 12%) levels decreased in a dose dependent manner. The decrement of cholesterol level was found statistically significant in medium and high dose groups while the testosterone concentration decreased nonsignificantly. The plasma luteinizing hormone (LH), gonadotropin-releasing hormone (GnRH) and inhibin B (INHB) hormone levels increased significantly in the low and medium dose groups (≥ 2%, *p* < 0.05) as compared to the control group. The follicle-stimulating hormone (FSH) level also increased significantly in all the treatment groups (≥ 1.57 fold, *p* < 0.0005). However, it was observed that the plasma INHB levels declined 11% at the high dose of acetamiprid group.

The antioxidative parameters (Reduced glutathione (GSH) and total antioxidant status (TAS)) was found to decrease significantly in plasma and testes tissues in a dose-dependent manner (*p* < 0.05). Additionally, the oxidative parameters (Malondialdehyde (MDA) and Total oxidant status (TOS) of plasma and testes tissue levels were observed to increase significantly in a dose dependent manner (*p* < 0.05) (Table 3). The parameters were dramatically changed by 35 mg/kg acetamiprid treatment.

**Table 3:** Hormones and oxidative stress parameters at the end of subchronical administration of oral acetamiprid in Sprague Dawley rats.

Plasma levels (mean ± SEM)				
	Control group (n:10)	Low dose group (n:11)	Medium dose group (n:12)	High dose group (n:12)
Testosteron (ng/mL)	2.32 ± 0.23	1.80 ± 0.49	1.65 ± 0.29	1.52 ± 0.17
LH (mIU/mL)	11.07 ± 0.57	12.65 ± 0.58*	13.13 ± 0.17*	11.86 ± 0.20
FSH (IU/L)	7.19 ± 0.56	11.87 ± 0.38***	14.27 ± 0.61***	11.30 ± 0.22***
GnRH (ng/L)	194.89 ± 11.88	207.72 ± 7.49	209.845 ± 0.95	200.67 ± 8.92
INHB (ng/mL)	8.25 ± 0.28	9.18 ± 0.16	10.070 ± 0.30*	7.35 ± 0.53
Cholesterol (mmol/L)	5.46 ± 0.53	4.80 ± 0.34	4.39 ± 0.35*	4.20 ± 0.26*
GSH (mmol/mg protein)	0.47 ± 0.04	0.16 ± 0.01**	0.22 ± 0.01***	0.21 ± 0.01***
MDA (ng/mg protein)	0.56 ± 0.01	0.82 ± 0.002***	1.03 ± 0.01***	1.46 ± 0.06***
TAS (U/mL)	34.67 ± 2.10	16.48 ± 0.78***	14.23 ± 0.65***	10.24 ± 0.82***
TOS (nmol/mL)	2.73 ± 0.26	2.82 ± 0.09	2.97 ± 0.12	3.68 ± 0.10***
Tissue levels (mean ± SEM)				
GSH (mmol/mg protein)	2.37 ± 0.006	1.99 ± 0.12***	1.68 ± 0.02***	1.52 ± 0.03***
MDA (ng/mg protein)	6.57 ± 0.46	6.87 ± 0.40	9.81 ± 1.007*	11.30 ± 0,52***
TAS (U/mL per g protein)	46.53 ± 1.61	39.63 ± 1.26*	38.03 ± 1.28***	30.54 ± 1.26***
TOS (nmol/mL per g protein)	12.88 ± 0.03	13.28 ± 0.42	13.73 ± 0.22	14.93 ± 0.20***

Data were shown as mean ± standard error of the means (SEM). *p < 0.05, **p < 0.005, ***p < 0.0005. LH: Luteinizing hormone, FSH: Follicle-stimulating hormone, GnRH: Gonadotrophin releasing hormone, INHB: Inhibin B, GSH: Glutathione, MDA: Malondialdehyde, TAS: Total antioxidant status, TOS: Total oxidant status.

### Effects of acetamiprid on histopathology of testes

**Figure 1:**
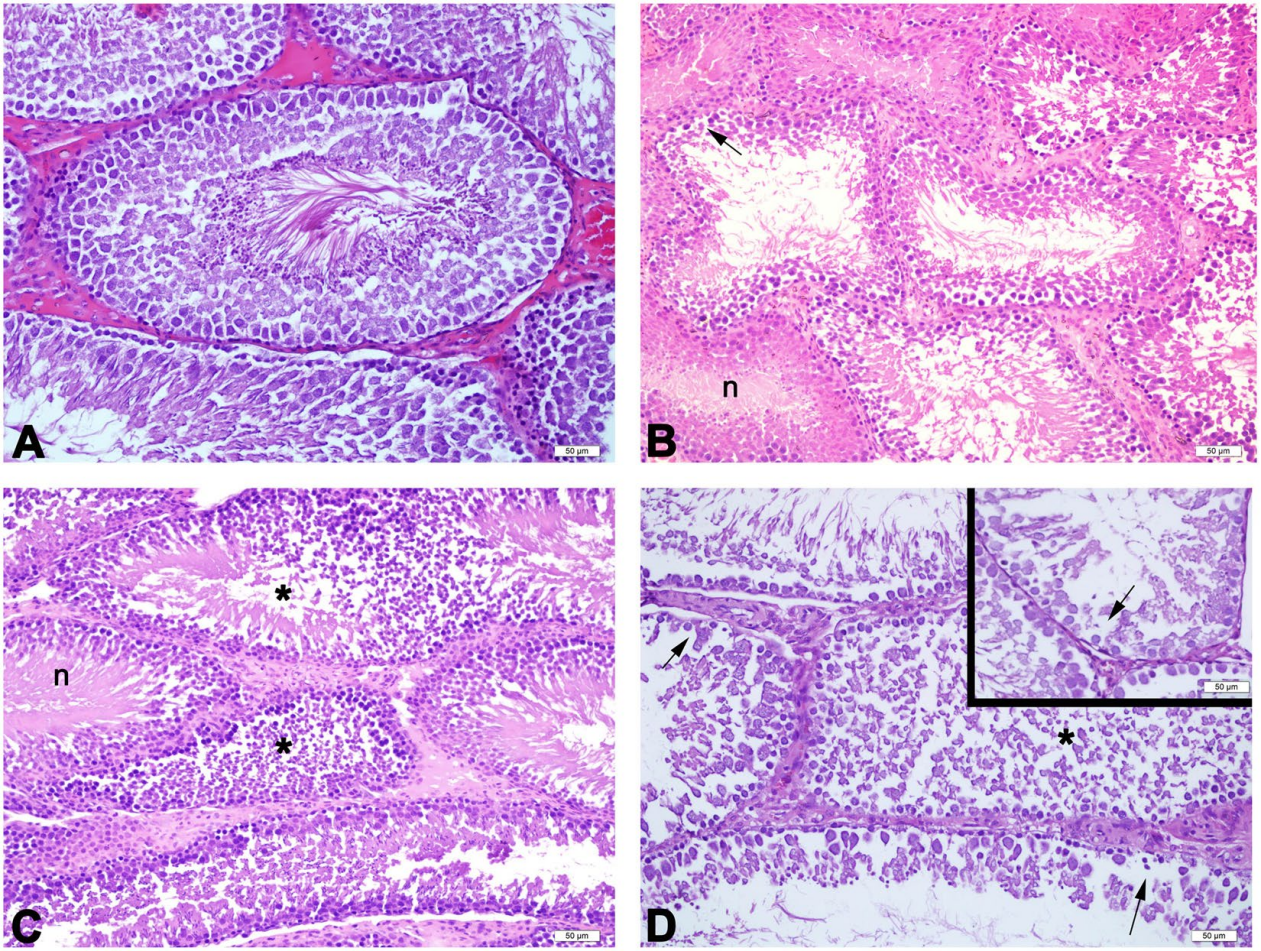
Representative light micrographs of experimental groups. Normal testis morphology with regular seminiferous tubules are seen in the control group (**A**). Normal seminiferous tubules (n) with a regular germinal epithelium and vacuolation (arrow) in the low-dose group (**B**). Some seminiferous tubules with immature cells (*) in their lumen as well as regular seminiferous tubules (n) in the medium-dose group (**C**); immature cells (*) in the lumen of some seminiferous tubules and vacuolation (arrow, inset) in the germinal epithelium in the high-dose group (**D**) are seen. H&E staining. Scale bars 50 µm.

**Figure 2:**
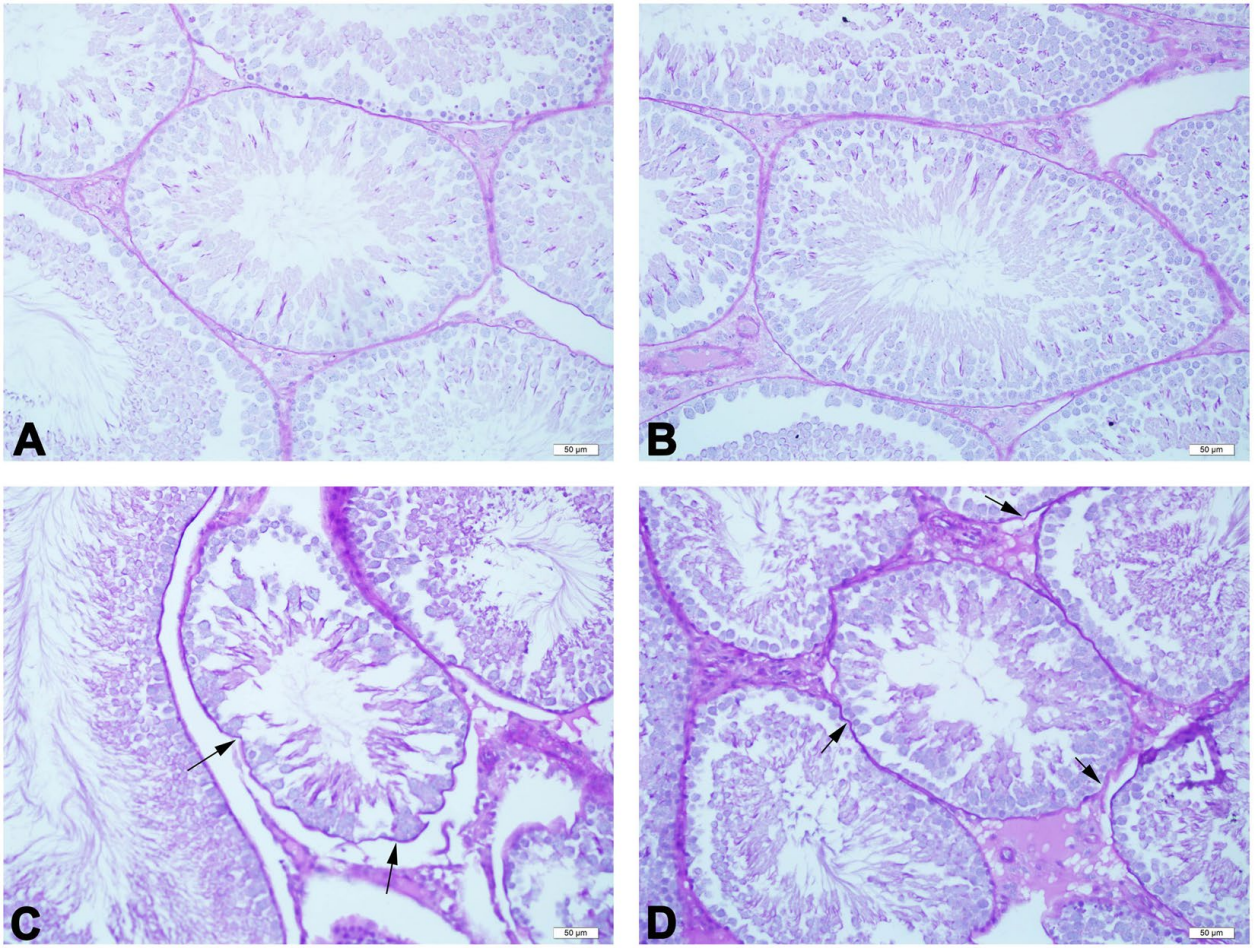
Representative photomicrographs of experimental groups. The regular basement membran of seminiferous tubules are seen in the control (**A**) and low-dose groups (**B**). Irregular and ondulated basement membrane (arrow) of the semniferous tubules are seen in the medium- dose (**C**) and high-dose groups (**D**). PAS reaction. Scale bars 50 µm.

In the control group, normal testicular morphology with regular spermatogenic cells and seminiferous tubules with regular basement membrane was observed. In the low dose group (score 7.6) vacuole formation was observed in the germinal epithelium of some tubules. Regular basement membrane was observed in this group. In the medium-dose group (score 7.9), immature cells were observed in many tubule lumens. Decrease number of spermatogenic germ cells, damaged spermatogenic cells with vacuole formation and luminal immature cell rashes, were observed in a large number of seminiferous tubules in the high-dose group (score 8.1). Irregular and undulating basement membrane was observed in medium-dose and high-dose groups (Figs. 1 and 2). Seminiferous tubule score was decreased significantly all the treatment groups (*p* ≤ 0.01) as compared to the control group (Table 4).

**Table 4:** Histopathological scoring, proliferation index and apoptotic index in the testes of Sprague Dawley rats treated with oral acetamiprid.

	Control group (n:10)	Low dose group (n:11)	Medium dose group (n:12)	High dose group (n:12)
Seminiferous tubule score	9.8	8.1^##^	7.9^##^	7.6^##^
Proliferation index	42.0	37.1^##^	30.5^##^	24.8^##^
Apoptotic index (%)	0	10.6	26.3^#^	57.5^##^

*p < 0.05, ^#^*p* < 0.1, ^##^*p* < 0.01.

**Figure 3:**
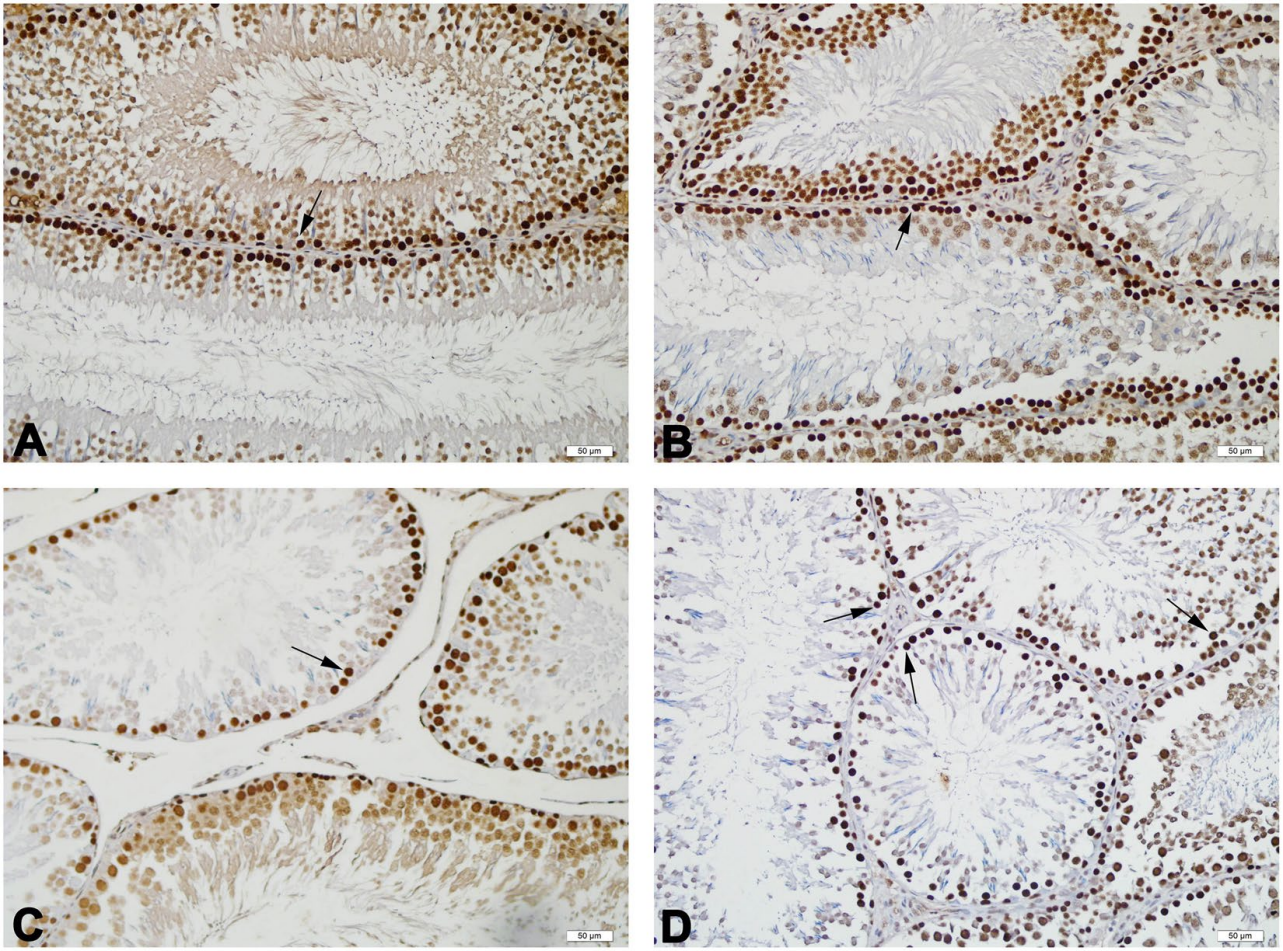
Representative photomicrographs of PCNA stained testis tissues of the experimental groups. PCNA-positive cells (arrow), numerous in the control (**A**) and low (**B**)-dose groups, moderately decreased in the medium (**C**)-dose group, and severely decreased in the high (**D**)-dose group, are observed. Scale bars 50 µm.

Proliferative cell nucleus antigen (PCNA)-positive cells were observed as dark brown in the seminiferous tubules of all groups. were observed (Fig. 3). Proliferation index was significantly decreased in all the treatment groups (*p* ≤ 0.01) as compared to the control group (Table 4).

**Figure 4:**
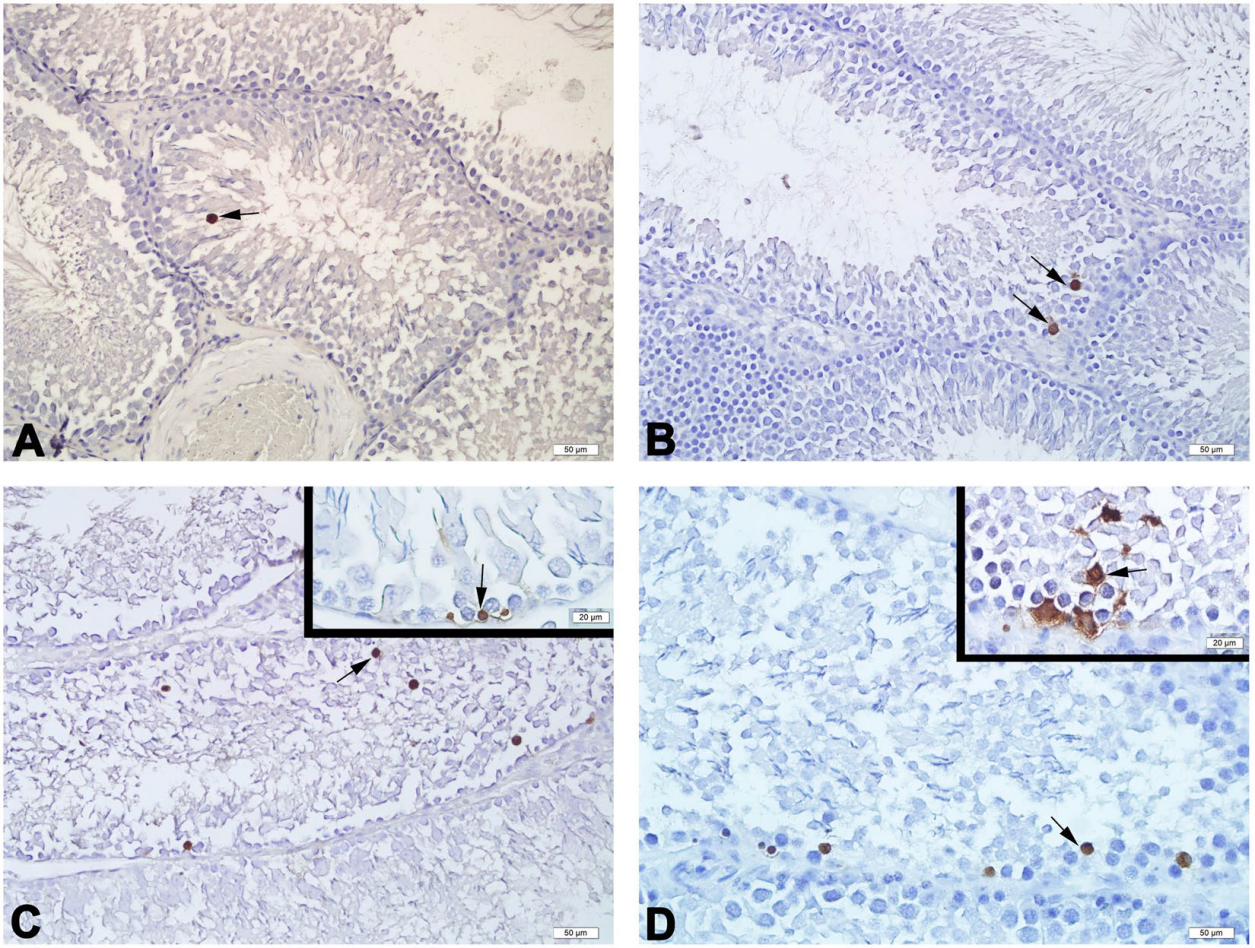
Representative photomicrographs of TUNEL stained testis tissues of the experimental groups. A few number of TUNEL-positive cells (arrow), in the control (**A**) and low (**B**)-dose groups, moderately increased in the medium (**C**)-dose group, and severely increased in the high (**D**)-dose group are observed. TUNEL. Scale bars 50 µm.

Terminal deoxynucleotidyl transferase (TdT) dUTP nick-end labeling (TUNEL)-positive cells in the seminiferous tubules of all groups were observed as dark brown (Fig. 4). The apoptotic index was significantly increased in the medium (*p* < 0.1), and high-dose groups (*p* ≤ 0.01) com*p*ared to the control group (Table 4).

## Discussion

To identify the toxic effects of acetamiprid which is one of the widely used neonicotinoid insecticide on the reproductive system, sperm morphology, oxidative stress parameters, apoptosis, changes in the body and testicular weights were evaluated. In the present study, body weight decreased minimally in all acetamiprid-treated groups at the last day of experiment comparing to the beginning of the experiment except for control group and this may be associated with acetamiprid treatment and there was no significant change in testicular weights among the treatment groups. Zhang *et al*.^7^ also reported acetamiprid reduced body, and testis weights, in Kunming male mice treated with 30 mg/kg acetamiprid orally during 35 days. Similarly, Devan *et al*.^8^ observed a decrease in testicular absolute organ weight, and an increase in absolute organ weights, which of these changes are statistically non-significant in Wistar rats treated with orally acetamiprid at doses of 27.5, 55 and 110 mg/kg body weight. In the same study, NOAEL value was found to be ≤ 55 mg/kg.

It has been demonstrated that acetamiprid caused to change in serum testosterone levels and seminiferous tubule diameter and thickness in albino mice exposed to 0.16 mg/ml, and 0.22 mg/ml, by adding acetamiprid to drinking water for four weeks^16^. According to a study which was conducted by Rasgele^17^, acetamiprid did not increase the sperm abnormalities at the doses of 0.62, 1.25, and 2.50 μg/mL after 24 and 48 hours on in *Mus musculus* mice. A study conducted by Kong *et al*.^18^ demonstrated that acetamiprid increases LH levels and decreases testosterone levels and sperm count in Sprague Dawley rat treated with 10 mg/kg and 30 mg/kg dose. Zhang *et al*.^7^ also showed 30 mg/kg acetamiprid treatment decreased intact acrosome rate of sperms and testosterone level in Kunming male mice. In a study conducted by Mosbah *et al*.^19^, acetamiprid has been reported to increase in body weight and decline in testis weights, sperm count, plasma testosterone level, sperm motility in Wistar rats dosed by 27 mg/kg acetamiprid by gavage (5 days per week) for 45 days. In the present study, similar to Mosbah *et al*.^19^ we also found that acetamiprid induced sperm abnormalities and decreases sperm concentration and plasma testosterone levels in a dose dependent manner. Since testosterone which is main circulating androgen is synthesized from cholesterol^20^, the decrease of the plasma testosterone may result from decrease of plasma cholesterol level Eacker *et al*.^21^, and it was found that plasma cholesterol level declines significantly in a dose dependent manner. Besides, acetamiprid may induce oxidative stress in Leydig cells and can play a role in decreasing testosterone secretion^19,22^.

In addition to testosterone, GnRH, FSH, LH, and INHB hormones have important roles in spermatogenesis. The balance of these hormones is necessary for the proper functioning of the testes^22^. Thus, plasma levels of the hormones were also evaluated. Acetamiprid was found to disrupt the hormonal balance in the present study. It has been reported that increase in serum FSH and LH levels, and decrease in serum testosterone and INHB levels has been detected in patients who have low sperm concentration, and this can be used as biomarker for the diagnosis of the infertility in the clinic^23,24^. In the current study, we demonstrated that acetamiprid caused to increase FSH and LH plasma levels in all treatment groups. Although sperm count diminished in a dose dependent manner, the rate of the increase of these hormones was found to be lower in the high dose group. Interestingly, the plasma INHB level increased at the low and medium dose group whereas it decreased at the high dose group.

It has been stated that acetamiprid leads to oxidative stress in many organs such as liver, kidney, brain of different species^8,25^. Oxidative stress has been shown to cause damage in DNA, proteins and lipids of sperm and also induce apoptosis. As it is known, these mechanisms might play a role in decrease of sperm count and impairment of sperm function^26^. It is known that oxidative stress is one of the common reason of testicular dysfunction which leads to infertility^27^. Reactive oxygen species (ROS) production in the testis leads to oxidative damage and destroys steriodogenesis and spermatogenesis^28^. Some studies showed acetamiprid induced lipid peroxidation and reduced antioxidant enzymatic activity in the testes^12,18^. In the current study, acetamiprid led to lipid peroxidation, GSH depletion, increase in TOS, and decrease in TAS in testis homogenates in a dose dependent manner. These results were also confirmed in the plasma. However, these parameters were more affected in testis homogenate than plasma. This can be associated with high potential of ROS formation in the cells that use molecular oxygen in steroid biosynthesis in testes^29^. Oxidative stress also affects the regulation of proliferation, apoptosis and transcription in the testis^30^. Thus, acetamiprid-induced oxidative stress might result in reduced sperm count, increased sperm abnormalities and apoptosis induction in the study. Moreover, it has been stated testosterone might have antioxidant and antiapoptotic function in testes and protect sperm from DNA damage^31,32^ and decreased testosterone level may augment acetamiprid-induced oxidative stress in the present study. Besides, it has been stated INHB is released more in oxidative stress^32^. However, INHB level decreased nonsignificantly at high dose treatment group which we observed extensive oxidative stress compared to control group. This might be associated with dramatically increased apoptosis in testes at the high dose group.

As it is well known, the alterations of apoptosis have been involved in pathology of the many diseases such as cancer and neurodegenerative diseases^33^. It has been also showed that cell injury in testis may result in apoptosis^34^. In the present study, TUNEL cell count and apoptotic index were used to evaluate apoptotic cells. The number of apoptotic cell-containing seminiferous tubules was higher in the exposure groups than in the control group. The apoptotic index levels were 10.6, 26.3 and 57.5, respectively, in the low-, medium- and high-dose groups. Increased apoptosis may be a consequence of acetamiprid-induced oxidative stress. Histopathological examination revealed apoptotic cells in the tubules of all exposed groups, mostly in spermatogonia and primary spermatocytes. The number of apoptotic cells increased in a dose-dependent manner. According to the results of seminiferous tubule scoring and histopathological examination, the basement membranes of the seminiferous tubules were found to be regular in the control and low-dose exposure groups. In the medium- and high-dose exposure groups, the presence of basal membranes showing irregular and undulate structure in many seminiferous tubules was detected. In the high-dose group, a decrease in spermatogenic germ cell counts, spermatogenic cells with vacuole formation and luminal immature cell rashes were observed in most seminiferous tubules.

Spermatogenesis perturbation and weak sperm were seen in some seminiferous tubules of the Wistar rats treated with 27 mg/kg oral acetamiprid for 45 consecutive days. The presence of pelleted cell particles in the lumen, tubulous atrophy, disorganisation and degenerative direction of the seminal epithelium were observed^35^.

According to a study, degeneration of spermatogonia and edematous changes in seminiferous tubules and occlusion of blood vessels in the interstitial space have been reported in Swiss albino male mice treated with 2.3 mg/kg and 4.6 mg/kg acetamiprid for 30, 60 and 90 days^36^. In the present study, the proliferation index decreased in a dose-dependent manner, particularly in the high-dose exposure group by PCNA immunohistochemical technique.

In conclusion, acetamiprid was found to have potentially toxic to the male reproductive system on Sprague Dawley rats at the dose of 35 mg/kg. Underlying mechanisms of the toxic effects can be associated with oxidative stress and apoptosis in the testes. This data emphasizes the importance of taking precautions against environmental and occupational exposure to agricultural drugs. However, further mechanistic studies are needed to understand the molecular mechanisms of the acetamiprid induced reproductive toxicity.

## Material and Method

### Animals and their synchronization

A total of 48 (8–10 weeks old) male Sprague-Dawley rats with body weights of 300–350 g were obtained from Istanbul University Aziz Sancar Institute of Experimental Medicine and were divided into 4 groups, control group (n = 10), low dose group (n = 11), medium dose group (n = 12) and high dose group (n = 12). Rats were housed in polystyene standard cages with 4–5 animals in each, maintained at 21–23 °C and humidity (55 ± 5%) at Istanbul University Faculty of Pharmacy Animal Facility Unit (EDEHAB). Rats synchronized for a 12 h light/12 h dark cycle (LD 12:12). Standard pellet chow and tap water provided *ad libitum* throughout the experiment. Acetamiprid administration was performed during light span and commenced at 10:00 AM equal to 3 hours after light onset i.e. HALO-3 to prevent time dependent differences. The study was approved by Istanbul University Local Ethics Committee of Experimental Animals (IUHADYEK; 2016/35) and all experiments were performed in accordance with Istanbul University Animal Experiments Local Ethics Committee Guideline^37,38^.

### Chemicals and reagents

Acetamiprid (97% of purity) was kindly gifted from Hektaş Ticaret T.A.Ş. (Istanbul, Turkey) and freshly suspended in an aqueous solution of 0.5% methylcellulose (MC) on each study day. Acetamiprid was orally administered to the rats in a fixed fluid volume (4 mL/kg body weight)^37^.

Sperm morphology was evaluated using phosphate-buffered saline (PBS 10×), Dulbecco’s Modified Eagle Medium/Nutrient Mixture F-12 (DMEM-F12), and fetal bovine serum (FBS) (Multicell Wisent, Quebec, Canada). For histological evaluation of testes, polylysine coated microscope slides and covers (Thermo Scientific, Munich, Germany) and microtome blades (Cologne, Germany), analytical grade chemicals (Sigma Aldrich, Munich, Germany and Merck Millipore, Munich, Germany) were used, while plastic wares from Eppendorf (Nijmegen, Netherlands) and Nest Biotechnology (Munich, Germany).

### Experimental design

The threshold levels have been reported for the toxicity profiles of acetamiprid in female/male Sprague Dawley rats. No observable adverse effects level (NOAEL) and medium lethal dose (LD_50_) of acetamiprid were 7.1 mg/kg and 217 mg/kg, respectively^39^. According to the European Food Safety Authority (EFSA) 2016 data, reproductive NOAEL was 51 mg/kg^40^. The NOAEL of acetamiprid determined was 38.7 mg/kg mg/kg by two generation study in rats^41^. In our preliminary study, oral dose of 50 mg/kg of acetamiprid resulted in 25% of animal death. Accordingly, the acetamiprid dose groups were divided as highest dose group (35 mg/kg/day), medium dose group (25 mg/kg/day), and the low dose group (12.5 mg/kg/day). Acetamiprid was administered to rats during 90 days, by using oral gavage used for the rats. The control group was given only vehicle i.e., 0.5% MC.

At the end of the 90^th^ day, the rats were exposed to inhalation anaesthesia for very short time. Then, they were sacrificed by removing large volume of blood from the orbital veins and immediately incised from abdominal to breast area. The blood samples were collected in EDTA tubes for hormone and biochemical analysis. The testes and epididymis were dissected, and sperms were collected from the epididymis. The left testis was homogenized in a sufficient amount of PBS (1×), and stored at −80 °C until biochemical analysis. The right testis was fixed in a sufficient amount of 10% neutral buffered formalin, and stored at room temperature until histological examinations.

### Body weight and testis weights

Body weights of all animals were measured (Sartorius, Mettler H20, Germany) three times a week and at the end of the experiment. Mortality and clinical signs e.g. posture, locomotor activity, were controlled every day. Testicles were removed on sacrifice and weighed (Precisa XB220A, Switzerland) for the toxicological evaluation (U.S. Enviromental Protection Agency 1996). The testes were also evaluated macroscopically to reveal any enlargement, shrinkage, gaps due to tissue loss, tissue softening, tissue foreign coloring, and altered content.

The epididymis was cleaned from peripheral structures carefully, and weights were recorded. The somatic index was calculated by employing the following formula:

$${\rm{Organ}}/{\rm{Body}}\,{\rm{weight}}\,{\rm{index}}={\rm{Organ}}\,{\rm{weight}}/{\rm{Body}}\,{\rm{weight}}\times 100$$

### Epididymal sperm counting and morphology

#### Sperm collection and count

The caudal epididymis was minced with scissors in petri dish *containing* DMEM-F12 medium with 10% PBS to release sperm. The suspension was centrifuged at 2,230 rpm for 3 min. The supernatant was centrifuged again and then was added to trypan blue (1:1, v/v). The mixture was spread on the Thoma slide. The sperms were counted under a Leica microscope (Leica Microsystems Co., Wetzlar, Germany) at 20X magnification.

#### Sperm morphology

To assess sperm morphology, one drop of the epididymis suspension was spread on the slides and allowed to dry at room temperature. The morphological examinations of spreading samples were completed within one week. The samples were prepared in accordance with the Diff-Quick staining protocol (ADR, Istanbul, Turkey), and were carried out with 3 spreads for each sample and 200 sperm were counted for each preparation. The sperms were classified as normal, headless, detached head, flattened head, pinhead, bent neck, bent tailed, coiled tail and multiple abnormalities^42,43^.

### Hormone and biochemical analysis in plasma and testicular tissue

The blood samples were centrifuged at 3000 rpm for 20 min −4 °C (Hettich Universal 32 R Germany). The plasma was separated and kept in −20 °C refrigerator (Arcelik 2041, Turkey). The quantitative determination of plasma cholesterol, testosterone, LH, FSH, GnRH and INHB was carried out in plasma by commercial kits according to the manufacturer’s instructions (Elabscience, Wuhan, China, and SunRed, Shanghai, China).

The testes tissues were homogenized in PBS (1:5, w/v) and kept at −80 °C (Daihan-Scientific Wisecry, South Korea). Biochemical parameters GSH, MDA, TAS and TOS indicating oxidative damage were measured by enzyme-linked immunosorbent assay (ELISA) commercial kits according to the manufacturer’s instructions (Elabscience, Wuhan, China, and SunRed, Shanghai, China). The optical density (OD) was measured at 450 nm, with a reference wavelength of 620 to 630 nm using an Epoch microplate spectrophotometer (BioTek Inc., Bad Friedrichshall, Germany).

### Histological examination of testicular tissue

For the histological examination, the right testis was fixed in 10% neutral buffered formalin, dehydrated in a series of graded alcohol and embedded in paraffin^44,45^. Hematoxylin and eosin (H&E) staining was applied to the middle part of testis taken from paraffin blocks at a thickness of 4 μm for histopathologic analysis, and periodic acid Schiff (PAS) staining was performed to evaluate changes of seminiferous tubule basement membrane. The evaluation was performed on each of the 30 seminiferous tubules with a 20X microscopic magnification. The first evaluated seminiferous tubule was randomly selected, and the section was shifted clockwise to evaluate the others. Histopathologic scoring was assessed by the modified Johnson scoring method (Table 5)^46^.

**Table 5:** Modified Johnson Scoring Method^22^.

Score	Histological Findings
10	Full spermatogenesis
9	Slightly impaired spermatogenesis, many late spermatids, disorganized epithelium
8	Less than five spermatozoa per tubule, few late spermatids
7	No spermatozoa, no late spermatids, many early spermatids
6	No spermatozoa, no late spermatids, few early spermatids
5	No spermatozoa or spermatids, many spermatocytes
4	No spermatozoa or spermatids, few spermatocytes
3	Spermatogonia only
2	No germinal cells, Sertoli cells only
1	No seminiferous epithelium

#### PCNA immunohistochemistry

The PCNA immunohistochemistry were done by using commercial kit from Invitrogen (Carlsbad, California, USA). For PCNA immunohistochemistry, half of the right testes were fixed for 72 h in 10% neutral buffered formalin. The paraffin sections with a thickness of 4 μm, which were collected on the positively charged slides, were done according to previous study^47^. Randomly selected, twenty seminiferous tubules were examined in the stained sections. The cells with brown nuclear staining were accepted PCNA positive and all of the stained and nonstained germ cells were counted. The ratio of PCNA positive cells to the total number of germ cells, “PCNA index,” was calculated for each seminiferous tubule. The average PCNA index in each case was obtained by dividing the sum of all PCNA indices by the number of seminiferous tubules in which the calculation was carried out.

$${\rm{Proliferation}}\,{\rm{index}}=({\rm{X}}1+{\rm{X}}2+{\rm{X}}3+\ldots \ldots +{\rm{X20}})/20$$

#### Detection of apoptosis by TUNEL assay

TUNEL assay were done by using ApopTag Plus Peroxidase *In Situ* Apoptosis Kit (Millipore, Massachusetts-USA) according to manufacturer’s protocol with slight modifications. Twenty seminiferous tubules were examined in the stained sections. The seminiferous tubules containing 3 or more apoptotic cells were counted as TUNEL. The apoptotic index was calculated by the following formula:

$${\rm{Apoptotic}}\,{\rm{index}}={\rm{number}}\,{\rm{of}}\,{\rm{TUNEL}}+{\rm{cells}}/{\rm{total}}\,{\rm{number}}\,{\rm{of}}\,{\rm{seminiferous}}\,{\rm{tubules}}$$

### Statistical evaluation

Data were expressed as means ± standard error of the means (SEM) for each studied variable. Statistical analysis was performed using the Statistical Package for Social Sciences (SPSS, v.20, Chicago, IL). The statistical significance of differences between groups was validated with one-way analysis of variance (ANOVA) followed by post hoc Tukey test for intergroup comparisons. *p* < 0.1, *p* < 0.05, *p* < 0.01, *p* < 0.005, and *p* < 0.0005 were considered significant.
